# Novel Prognostic Index of High-Risk Prostate Cancer Using Simple Summation of Very High-Risk Factors

**DOI:** 10.3390/cancers13143486

**Published:** 2021-07-12

**Authors:** Hideya Yamazaki, Gen Suzuki, Koji Masui, Norihiro Aibe, Daisuke Shimizu, Takuya Kimoto, Kei Yamada, Takumi Shiraishi, Atsuko Fujihara, Koji Okihara, Ken Yoshida, Satoaki Nakamura, Haruumi Okabe

**Affiliations:** 1 Department of Radiology, and Urology, Graduate School of Medical Science, Kyoto Prefectural University of Medicine, 465 Kajiicho Kawaramachi Hirokoji, Kamigyo-ku, Kyoto 602-8566, Japan; gensuzu@koto.kpu-m.ac.jp (G.S.); mc0515kj@koto.kpu-m.ac.jp (K.M.); a-ib-n24@koto.kpu-m.ac.jp (N.A.); dshimizu@koto.kpu-m.ac.jp (D.S.); t-kimoto@koto.kpu-m.ac.jp (T.K.); kyamada@koto.kpu-m.ac.jp (K.Y.); takumi14@koto.kpu-m.ac.jp (T.S.); fujihara@koto.kpu-m.ac.jp (A.F.); okihara@koto.kpu-m.ac.jp (K.O.); 2 Department of Department of Radiology, Kansai Medical University, Hirakata 573-1010, Japan; yoshidaisbt@gmail.com (K.Y.); satoaki@nakamura.pro (S.N.); 3 Department of Radiology, Ujitakeda Hospital, Uji-City 611-0021, Japan; h-okabe@takedahp.or.jp

**Keywords:** very high-risk, T3b–4, Gleason 9–10, prostate cancer, brachytherapy

## Abstract

**Simple Summary:**

We explored the role of each very high-risk factor and found that simple summation of the number of very high-risk (VHR) factors (T3b–4 and Gleason score 9–10) is an easy and very high predictive power to separate VHR-2 (both T3b–4 and Gleason score 9–10) and others (VHR-1; T3b–4 or Gleason score 9–10, VHR-0; none of T3b–4 and Gleason score 9–10). The VHR-2 group showed a strikingly lower biochemical control rate and distant metastasis free survival rate than other groups, resulting in higher prostate cancer specific mortality than the VHR-1 and VHR-0 groups.

**Abstract:**

This study aimed to examine the role of very high-risk (VHR) factors (T3b–4 and Gleason score 9–10) for prognosis of clinically localized high-risk prostate cancer. We reviewed multi-institutional retrospective data of 1413 patients treated with radiotherapy (558 patients treated with external beam radiotherapy (EBRT) and 855 patients treated with brachytherapy (BT) ± EBRT. We introduced an index by simple summation of the number of VHR factors—VHR-0, VHR-1, and VHR-2. With median follow-up of 69.6 months, the 5-year biochemical disease free survival rate (bDFS), prostate cancer-specific mortality (PCSM), and distant metastasis-free survival (DMSF) rates were 59.4%, 7.65%, and 83.2% for the VHR-2 group, respectively; 86.7%, 1.50%, and 95.4% for the VHR-1 group, respectively; and 93.1%, 0.12%, and 98.2% for the VHR-0 group, respectively. The VHR-2 group had significantly worse bDFS, PCSM, and DMSF than the VHR-0 (hazard ratios: 4.55, 9.607, and 7.904, respectively) and VHR-1 (hazard ratios: 1.723, 2.391, and 1.491, respectively) groups. The VHR-2 group could be identified as a super high-risk group compared with other groups, and could be a good candidate for clinical trials using multimodal intensified treatments. Simple summation of the number of VHR factors is an easy and useful predictive index for bDFS, PCSM, and DMSF.

## 1. Introduction

Risk stratification in newly diagnosed prostate cancer is an important diagnostic process for selecting an optimal management approach for both physicians and patients. The most widely used risk classification system is the National Comprehensive Cancer Network (NCCN) [1]. The high-risk category was defined as biopsy Gleason score sum ≥ 8, prostate-specific antigen (PSA) level > 20 ng/mL, or clinical stage ≥T3a, which helps identify patients who have a high risk of recurrence and progression after treatment [1].

There is heterogeneity in the high-risk group: the rates of 10-year freedom from biochemical recurrence (bDFS) after surgery ranged from 25 to 68% [2]. To meticulously select patients for adequate treatment, high-risk prostate cancer was subdivided into the very high-risk (VHR) group, considered to have the worst prognosis, including those with primary Gleason score = 5, >4 biopsy cores with a Gleason score of 8–10, or clinical stage T3b–T4 [1], which comprised a surgical cohort of 753 high-risk patients [2]. The influence of each VHR factor in patients after radiotherapy and the best separation system is unclear because there is insufficient information to determine the outcome of patients with VHR factors after radiotherapy and they are treated with the same protocol as the high-risk group [3,4,5,6]. Therefore, we tried to examine the importance of the VHR factors (T3b–4 and Gleason score 9–10) for radiotherapy and developed an easy identification index by simply summing the number of VHR factors while preserving the point-of-care clinical applicability of the existing NCCN risk strata.

To analyze a large cohort, we used freely available public data on high-dose rate brachytherapy (HDR-BT) boost and external beam radiotherapy (EBRT) [7], including low-dose rate (LDR-BT) ± EBRT [8] and intensity-modulated radiotherapy (IMRT) performed in our institution [9]. Therefore, we aimed to examine the role of VHR factors for prognostication of clinically localized high-risk prostate cancer.

## 2. Materials and Methods

### 2.1. Patients

We retrospectively examined the data of patients treated with BT + EBRT (822 patients treated with HDR-BT boost identified from open data for public use and 33 patients treated with LDR-BT ± EBRT at Kyoto Prefectural Medical School) [7,8] and EBRT (417 patients treated with EBRT identified from open data and 141 patients treated with intensity modulated radiotherapy [IMRT] at Uji Takeda Hospital) [7,9] (Table 1). Patients treated with BT ± EBRT or EBRT; with clinical TNM stage T1–T4, with N0M0 disease with histology-proven adenocarcinoma; and with available and accessible data on pretreatment PSA (initial PSA [iPSA]) level, Gleason score sum, and T classification were eligible for this study. Patients were staged and were eligible if they were categorized as high-risk patients according to the NCCN risk classification—they have at least one of those high risk factors: (i) T3–4, (ii) Gleason score = 8–10, or (iii) PSA level > 20 ng/mL [1]. In general, pretreatment evaluation included clinical history, physical examination, blood laboratory findings, pelvic computed tomography (CT), and a bone scan. Magnetic resonance imaging (MRI) was recommended on request [6]. We created a simple index by summing the number of VHR factors—VHR-0: no VHR; VHR-1: Gleason score = 9–10 or T3b–T4; VHR-2: Gleason score = 9–10 and T3b–T4. We used cut-off value at Gleason 9–10 because Kuban et al. reported the importance of a Gleason score of 9 or 10 as a predictive factor for prostate cancer-specific mortality (PCSM) [10]. In addition, the new International Society of Urological Pathology grading system separates Gleason score 9–10 disease as a distinct entity with poorer outcomes [11].

PSA failure was defined using the Phoenix definition (nadir, +2 ng/mL). Prostate cancer specific mortality (PCSM) was defined based on either clinical documentation or inclusion of prostate cancer as a primary cause of death on a death certificate. Patients were classified as having distant metastasis when they had imaging evidence of lesions that were clinically or pathologically diagnosed as metastatic. Typically, imaging to detect distant metastasis was performed at the time of PSA failure or for subsequent PSA increases after an initial PSA failure. Outcomes of interest included bDFS, PCSM, OS, and MFS, which were defined by intervals from the start of radiotherapy to PSA failure, distant metastasis, PCSM, and death, respectively. All patients from Kyoto Prefectural Medical School and Uji Takeda Hospital provided written informed consent, and patients whose information were included in the public data provided an informed consent during the process of building public data. This study was conducted in accordance with the Declaration of Helsinki and was approved by the institutional review board of Kyoto Prefectural University of Medicine (ERB-C-1403).

### 2.2. Treatment Planning 

#### 2.2.1. Brachytherapy with or without External Beam Radiotherapy (BT ± EBRT)

BT ± EBRT groups consist of high dose rate brachytherapy (HDR-BT) with external beam radiotherapy (EBRT) or low dose rate brachytherapy (LDR-BT) with or without EBRT. We used multi-institution data from an open data source for HDR-BT [7], and details of the treatment have been described elsewhere [6]. All HDR-BT treatments used a combination of HDR-BT (median dose 31.5 Gy, range, 10.5–31.5 Gy) and EBRT in various fractions (Supplemental Table S1). The median fraction size of HDR-BT was 6.3 Gy (range, 5–11 Gy), while that of EBRT was 3 Gy (range, 2–3 Gy). For details of the treatment for LDR, the implant technique was previously described in detail [8]. We performed permanent intraoperative Iodine-125 implantation. We used combination therapy for patients with T3 disease or Gleason score sum ≤ 8 or Gleason score sum of 7 (4 + 3), but not for those with Gleason score sum of 7 (3 + 4) [8]. The prescription dose for the clinical target volume (prostate) was 145 Gy (LDR-BT alone) or 110 Gy (LDR-BT with 40 Gy/20 fractions EBRT by three-dimensional conformal radiotherapy (3D-CRT); Supplemental Table S1. For LDR and almost HDR cases, we used localized CTV; prostate + base of seminal vesicle in EBRT. However, several institutions used whole pelvic radiotherapy for the initial part of EBRT. Please refer to Table S1. We used planned follow-up by PSA blood test carried out every 3 months for the first 2 years, and every 6 months thereafter.

#### 2.2.2. External Beam Radiotherapy (EBRT)

The EBRT group received conventional two-dimensional treatment, 3D-CRT, and IMRT. The details are shown in Table S1. Some EBRT data were obtained from a freely accessible dataset (*n* = 417) [7] and 141 image-guided IMRTs using helical TomoTherapy were performed at the Department of Radiology, Uji Takeda Hospital. The detailed technique of image-guided IMRT using helical TomoTherapy has been described elsewhere [9]. The prescribed dose was 74.8 Gy/34 fractions (2.2 Gy/fraction, *n* = 62) between June 2007 and 2009, with 95% of the planning target volume (PTV) receiving at the least prescribed dose (D95), and was reduced to 74 Gy/37 fractions (2 Gy/fraction, *n* = 79) for the high-risk and intermediate-risk groups from June 2009 to September 2013 [9].

### 2.3. Statistical Analysis

StatView 5.0 and EZR stat package were used for statistical analyses [12]. EZR stat package was used to competing risk analysis (Gray analysis and Fine–Gray model). Percentages were analyzed using chi-square tests and Student’s *t*-tests were used for normally distributed data. Mann–Whitney U-tests and Kruskal–Wallis test for skewed data (i.e., PSA value) were used to compare means or medians. The Kaplan–Meier method was used to analyze the biochemical disease free survival rate (bDFS), distant metastasis free survival (DMSF), overall survival (OS), and Gray analysis for prostate cancer-specific survival rate (PCSM), and comparisons were made using log-rank tests or Gray analysis. Cause-specific manner (died of other cause of cancer was assigned as a censor) was applied to the bDFS, OS, and DMSF and competing risk analysis for PCSM. Cox’s proportional hazard model for bDFS, DMSF, and OS, and the Fine–Gray model for PCSM, were used for uni- and multivariate analyses. *p* < 0.05 was considered statistically significant.

## 3. Results

### 3.1. Patient and Tumor Characteristics

All 1413 patients with high-risk prostate cancer were treated with either BT ± EBRT (*n* = 855) or EBRT (*n* = 558). The median age was 71 years (range, 60–89 years). The median value of iPSA was 20.5 ng/mL (range, interquartile range = 2682−1454 ng/mL, 9.86–39.4 ng/mL). The clinical characteristics of the patients are presented in Table 1. The median follow-up duration for the entire cohort was 69.6 (range: 2–177) months, with a minimum of 1 year for surviving patients or until death.

Table 2 shows the patient characteristics of the VHR-0, VHR-1, and VHR-2 groups. The VHR-2 group tended to be treated with more EBRT and ADT than the VHR-0 and -1 groups. The median value of iPSA was 20.7 ng/mL (range, interquartile range = 2682–1454 ng/mL, 9.2–34.68 ng/mL), 17.7 ng/mL (3.09–500 ng/mL, 9.45–43.9 ng/mL), and 36.5 ng/mL (5.3–391 ng/mL, 18.7–73.7 ng/mL) for the VHR-0, VHR-1, and VHR-2 groups, respectively.

### 3.2. Biochemical Disease-Free Survival Rate (bDFS)

**Figure 1:**
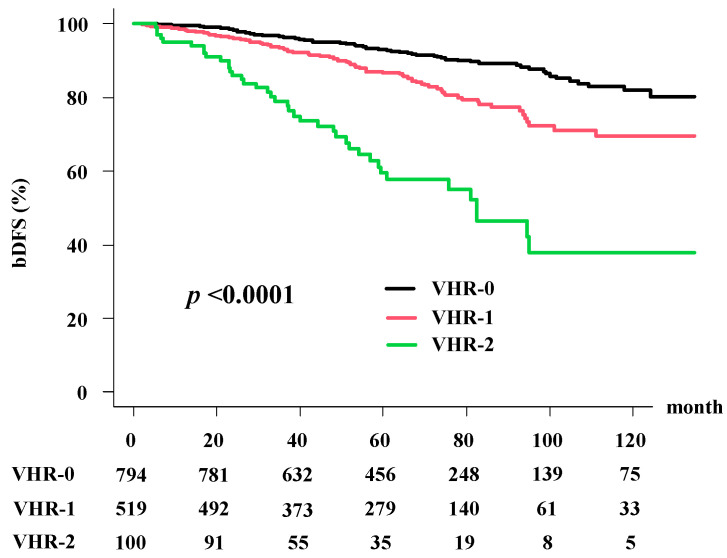
Biochemical disease-free survival rate (bDFS) according to the very-high risk (VHR) index.

The actuarial 5-year bDFS rate was 88.5% (95% confidence interval (CI): 86.2–82.2%) in all patients. The VHR-2 group showed worst bDFS (59.4%, 95% CI: 47.8–97.6%) at 5 years compared with the VHR-1 group (86.7%, 95% CI: 83.0–69.6%); and the VHR-0 group showed a 5-year bDFS of 93.1% (90.8–94.8%). There was a significant difference among the three groups (*p* < 0.0001; Figure 1).

As shown in Table 3, the predictors of bDFS in univariate analysis included treatment modality, T classification, Gleason score, baseline PSA level, and ADT duration. In multivariate Cox regression analysis (Table 4), treatment modality (BT ± EBRT) showed superior outcomes compared with EBRT; hazard ratio (HRa) = 0.447, 95% CI: 0.315–633, *p* < 0.0001) and VHR index (VHR-1 vs. VHR-0; HRa = 1.723, 95% CI: 1.256–2.362, *p* < 0.0001, VHR-2 vs. VHR-0; HRa = 4.55, 95% CI: 3.065–6.755, *p* < 0.0001) still had a significant influence on bDFS.

### 3.3. Distant Metastasis-Free Survival Rate (DMFS)

**Figure 2:**
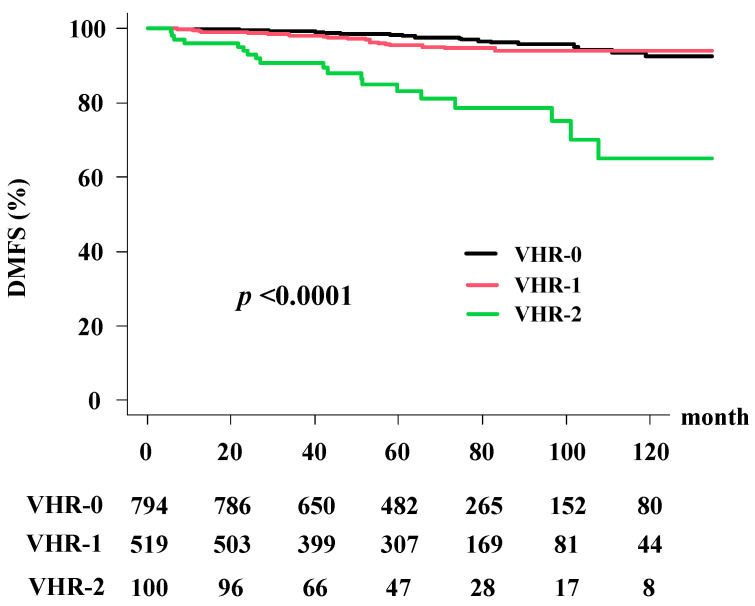
Distant metastasis-free survival rate (DMFS) according to the VHR index.

The distant metastasis-free survival rates (DMFS) were 96.1% (95% CI: 94.1%–97.1%) at 5 years and 90.8% (95% CI: 87.5%–90.2%) at 10 years (Figure 2). The VHR-2 group had the worst DMSF of 83.2% (95% CI: 72.9%–89.8%) at 5 years and 65.0% (95% CI: 46.0%–78.7%) at 10 years; the VHR-1 group was 95.4% (95% CI: 92.9%–97.1%) at 5 years and 94.0% (95% CI: 90.8%–96.1%) at 10 years; and the VHR-0 group was 98.2% (95% CI: 96.8%–99.0%) at 5 years and 92.4% (95% CI: 87.5%–95.4%) at 10 years. A significant difference was observed among the three groups (*p* < 0.0001; Figure 2).

As shown in Table 3, the predictors of DMSF survival rate on univariate analysis included treatment modality, T classification, Gleason score, baseline PSA level (−50 vs. 50<), and VHR index. In multivariate Cox regression analysis (Table 4), the VHR index between VHR-2 and VHR-0 (HRa = 7.904, 95% CI: 4.251–14.696, *p* < 0.0001) had a significant influence on OS.

### 3.4. Prostate Cancer-Specific Morality (PCSM)

**Figure 3:**
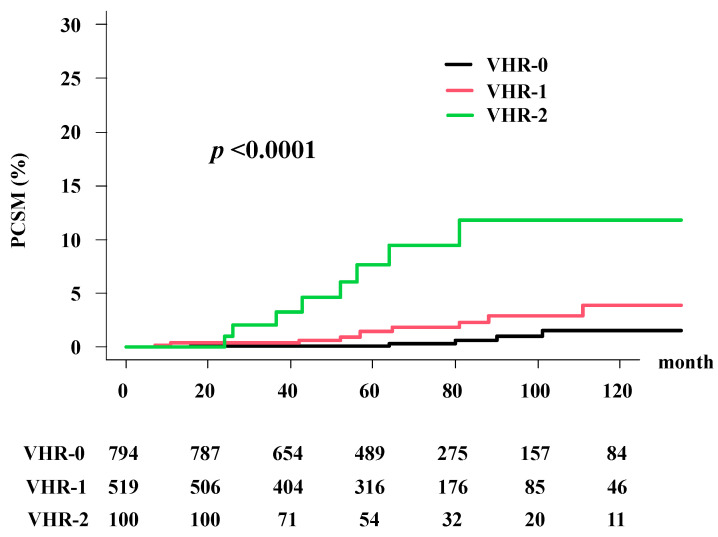
Prostate cancer-specific mortality (PCSM) according to the VHR index.

The cumulative incidence of prostate cancer-specific mortality (PCSM) was 1.14% (95% CI: 0.9–1.9%) at 5 years and 3.12% (95% CI: 1.5–3.8%) at 10 years in the total population. The VHR-2 group had PCSM rates of 7.65% (95% CI: 3.1–15.0%) at 5 years and 11.8% (95% CI: 5.9–21.2%) at 10 years; the VHR-1 group had PCSM rates of 1.5% (95% CI: 0.6–3.1%) at 5 years and 3.9% (95% CI: 1.8–7.5%) at 10 years; and the VHR-0 group had PCSM rates of 0.12% (95% CI: 0.0–0.7%) at 5 years and 1.5% (95% CI: 0.5–3.6%) at 10 years. Significant differences in PCSM were observed among the three groups (*p* < 0.0001; Figure 3).

As shown in Table 3, VHR indices showed a significant ability to stratify the risk of PCSM, and the predictors of PCSM in univariate analysis were T classification, Gleason score, and VHR index. In the multivariate Fine–Gray model (Table 3), the VHR remained a significant factor for PCSM between VHR-2 and VHR-0 (HRa = 9.067; 95% CI: 3.29–28.05, *p* < 0.0001).

### 3.5. Overall Survival Rate (OS)

**Figure 4:**
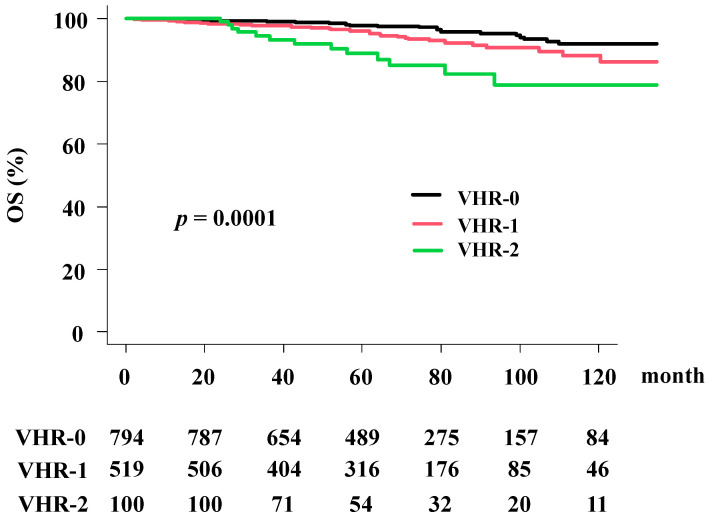
Overall survival rate (OS) according to the VHR index.

The overall survival (OS) rates were 96.4% (95% CI = 95.2–96.4%) at 5 years and 89.7% (86.2–96.2%) at 10 years in the total population. The VHR-2 group had worst OS rates of 88.9% (95% CI: 79.5–94.1%) at 5 years and 78.9% (95% CI: 64.7–87.9%) at 10 years; in the VHR-1 group, the rates were 95.9% (95% CI: 93.6–97.4%) at 5 years and 88.2% (95% CI: 82.1–82.3) at 10 years; and in the VHR-0 group, the rates were 97.7% (95% CI: 96.1–98.6%) at 5 years and 92.0% (95% CI: 87.6–94.9%) at 10 years. A significant difference was observed among the three groups (*p* = 0.0001; Figure 4).

As shown in Table 3, the predictors of OS in univariate analysis included treatment modality, T classification, Gleason score, baseline PSA level, and VHR. The results of the multivariate Cox regression analysis (Table 4) revealed that VHR (VHR-2 vs. VHR-0; HRa = 4.327, 95% CI: 2.206–8.487, *p* < 0.0001, VHR-1 vs. VHR-0; HRa = 1.88, 95% CI: 1.137–3.109, *p* = 0.013) remained significant factors for OS.

## 4. Discussion

Here, we have proposed an easy and useful index for a risk stratum that identifies men with worst oncological outcomes after radiotherapy for localized prostate cancer using a cohort of >1400 patients. These criteria may be beneficial for counseling individual patients regarding the treatment and prognosis of high-risk disease and the risk of requiring subsequent neoadjuvant, concurrent, or post-radiation therapies. The VHR criteria could also be useful as a risk stratification tool in future clinical protocols.

High-risk prostate cancer was subdivided into VHR groups in several ways. The NCCN used clinical stage T3b–T4 lesions, primary Gleason score = 5, or >4 biopsy cores with a Gleason score of 8–10 [1]. One of the initial studies that defined VHR was performed on the patients who underwent surgery [2] at Johns Hopkins, in which the VHR criteria used were multiple high-risk features, >4 biopsy cores with a Gleason score sum of 8–10, or primary Gleason score of 5 to create the risk factor groupings predictive for DMSF and PCSM. Then, they validated the role of VHR in three center cohorts, revealing HRa of 2.78 in DMSF, and 6.77 in PCSM to other NCCN high-risk men [13]. Narang et al. confirmed the role of VHR in high-risk patients undergoing radiotherapy plus androgen deprivation therapy at Johns Hopkins (HRa = DMSF: 2.49, PCMS: 3.19, and OS: 1.87, respectively) [14]. Our analysis confirmed the importance of VHR factors, in which VHR-2 showed the highest hazard risk for DMSF, PCSM, and OS compared with VHR-0 (HRa = 8.81, 11.99, and 4.644, respectively) and VHR-1 (HRa = 5.268, 2.359, and 2.896, respectively), and was distinctly better than the previous stratification system. The summation of the number of high risk factors has been explored in several studies including the above mentioned studies [2,4,6,13,14,15]. Wattson et al. reported a HRa of 4.8 for PCSM for those with at least two high risk factors compared with those with one high risk factor [16]. Our data may concur with this result; the VHR index also showed a significant difference. In addition, the VHR index showed an interesting characteristic—a significant difference (i.e., threshold) was only observed between VHR-2 and others in DMFS; therefore, it is useful to separate high-risk patients into VHR-2 and others. Rodrigues et al. reported ProCaRS classification [17]. They divided high risk patients by % core and iPSA value, and 40% of 5-year bDFS was found in extremely high-risk group. Although we could not compare our result directly to their outcome because we did not have information about % core, we could provide data for node 5 category (iPSA > 32.5 ng/mL, part of extremely high-risk group), in which 5-year bDFS was 83.5%. Therefore, Japanese patients tend to show a superior outcome to the Canadian population [17]. We used long term ADT, which could be one of the reasons of our good outcome compared with previous studies. Furthermore, good efficiency of ADT was found in Japanese men and is explained by the Japanese-specific high sensitivity to hormonal therapy [18].

The Gleason score is reported to be one of the most important factors for prognosis. Kuban et al. cited the importance of a Gleason score of 9 or 10, which was predictive of PCSM [10]. Sabolch et al. also reported that the presence of Gleason score of 5 on the biopsy specimen was the strongest prognostic factor for all clinical outcomes, including PCSM and OS after EBRT (≥75 Gy) with T1–T4 prostate cancer [19]. Our data partly concurred with their data because a significant threshold to separate PCSM with the highest hazard ratio was obtained between T3a and T3b and Gleason score sum 8 and 9, but not in iPSA (Table 3). Tsumura et al. reported similar results that stage T3b patients with grade group 5 may have a greater risk for PCSM [20].

BT ± EBRT showed superior efficacy in terms of bDFS compared with EBRT. This finding is typical because BT has a unique characteristic that allows it to deliver higher doses of radiation to the target lesion without excessive irradiation of the adjacent organs and is considered to be one of the best radiotherapy options [21]. Therefore, a number of studies and randomized controlled trials demonstrated the superiority of treatment by increasing the prescribed dose for localized prostate cancer in bDFS [22], especially with BT boost [22,23]. Our results are in line with the findings of a previous study, which indicated that BT improves bDFS. Furthermore, several studies found superior efficacy of dose escalation in terms not only of bDFS, but also of PCSM and OS [24,25,26]. At present, however, our data indicated that the combination of BT improved bDFS, but did not improve the PCSM or OS, and this discussion should be left for further studies.

Following advancements of treatment including long term ADT use, distant metastasis occurred only in 66 patients (4.6%) and PCSM in 26 patients (1.8%) out of more than 1400 patients. Of these, the lowest DMFS and highest PCMS ratio were found in the VHR-2 group compared with the other VHR groups, resulting in worst OS. Therefore, men with VHR-2 prostate cancer experience unusually aggressive oncologic outcomes and should be considered for intensive follow-up for metastasis using state-of-the-art technologies such as prostate-specific membrane antigen positron emission tomography scan [27] and/or adjuvant/earlier intervention with effective systemic therapy, such as docetaxel Abiraterone and Enzalutamide, in addition to longer periods of ADT use [28], that is, clinical trial settings using multimodal treatment.

There are several limitations to the present study. First, we could not examine the role of the biopsy core because the public database did not contain these data, and advancement in image-guided biopsy techniques made it impossible to assess for old data owing to its incompatibility with recent systems; high-grade tumor nodules were either undersampled or oversampled, and lacked a central pathologic review. Second, our study had limitations owing to its retrospective nature, limited follow-up time, and small sample size for reflecting the total prostate cancer patient population, which may limit the application of its findings. Thus, a longer follow-up with a larger sample is needed to obtain concrete conclusions. The authors should discuss the results and how they can be interpreted from the perspective of previous studies and of the working hypotheses. The findings and their implications should be discussed in the broadest context possible. Future research directions may also be highlighted.

## 5. Conclusions

A simple summation of the number of VHR factors is an easy and useful predictive index not only for bDFS, but also for PCSM and DMSF. These VHR-2 patients could be good candidates for more intense treatment with systemic agents.

## Figures and Tables

**Table 1:** Characteristics of patients.

Variables	Strata	Total	
		(*n* = 1413)	
		No. or Median (range)	(%)
Age		71 (60–89)	
T Category	≤2 3a 3b 4	583 587 215 28	(41%) (41%) (15%) (2%)
iPSA (ng/mL)	≤20 20< 50< 100<	684 474 151 104	(48%) (33%) (11%) (7%)
Gleason score	≤7 8≤ 9≤ 10≤	591 346 436 40	(42%) (24%) (31%) (3%)
Modality	EBRT BT ± EBRT	558 855	(39%) (60%)
Hormonal therapy	Yes	1348	(95%)
Duration	(Months)	40 (1–140)	
	No	65	(5%)
Neoadjuvant	Yes	1200	(85%)
Duration	(Month)	9 (1–92)	
Adjuvant	Yes	921	(65%)
Duration	(Month)	36 (1–134)	
Follow-up	(Months)	69.6 (2–177)	

BT = brachytherapy, EBRT = external beam radiotherapy, PSA = prostate-specific antigen.

**Table 2:** Comparison among three groups stratified with the very high-risk (VHR) index.

Variables	Strata	VHR-0 (*n* = 794)		VHR-1 (*n* = 519)		VHR-2 (*n* = 100)		*p*-Value
		No. or Median (Range)	(%)	No. or Median (Range)	(%)	No. or Median (Range)	(%)	
Age		70 (60–86)		71 (60–86)		70 (60–89)		0.02202
T Category	≤2	386	(49%)	197	(38%)	0	(0%)	**<0.0001**
	3a	408	(51%)	179	(34%)	0	(0%)	
	3b	0	(0%)	130	(25%)	85	(85%)	
	4	0	(0%)	13	(3%)	15	(15%)	
iPSA (ng/mL)	≤20	373	(47%)	281	(54%)	30	(30%)	**<0.0001**
	20<	306	(39%)	135	(26%)	33	(33%)	
	50<	74	(9%)	51	(10%)	26	(26%)	
	100<	41	(5%)	52	(10%)	11	(11%)	
Gleason score	−7	480	(60%)	111	(21%)	0	(0%)	**<0.0001**
	8	314	(40%)	32	(6%)	0	(0%)	
	9	0	(0%)	346	(67%)	90	(90%)	
	10	0	(0%)	30	(6%)	10	(10%)	
Modality	EBRT	273	(34%)	224	(43%)	61	(61%)	**<0.0001**
	BT ± EBRT	521	(66%)	295	(57%)	39	(39%)	
Hormonal Therapy	Yes	746	(94%)	503	(97%)	99	(99%)	**0.0088**
	(Months)	41 (1–112)		33 (2–140)		25 (4–128)		0.0777
	No	48	(6%)	16	(3%)	1	(1%)	
Neoadjuvant	Yes	669		438		93		
Duration	(Month)	10 (1–89)		9 (1–92)		8 (4–24)		0.0696
Adjuvant	Yes	526		334		61		
Duration	(Month)	36 (1–114)		36 (1–134)		36 (1–49)		**0.0171**
Follow-up	(Months)	68.2 (9–177)		67 (2–158)		62.9 (20.4–153)		0.2253
Prostate cancer-specific mortality(PCSM)	No Yes	787 7	(99%) (1%)	508 11	(98%) (2%)	92 8	(92%) (8%)	**<0.0001**
PSA failure	No	720	(91%)	435	(84%)	60	(60%)	**<0.0001**
	Yes	74	(9%)	84	(16%)	40	(40%)	
Overall survival	Alive	765	(96%)	486	(94%)	87	(87%)	**0.0002**
	Death	29	(4%)	33	(6%)	13	(13%)	
Distant metastasis	No	769	(97%)	497	(96%)	81	(81%)	**<0.0001**
	Yes	25	(3%)	22	(4%)	19	(19%)	

Bold values indicate statistically significance, NA; not available. BT = brachytherapy, EBRT = external beam radiotherapy. VHRF-0, -1, and -2 indicate no VHRF, one VHRF, and two VHRF.

**Table 3:** Uni-variate analysis for biochemical, overall, prostate cancer-specific, and metastasis-free survival rate using the Cox proportional hazards model or the Fine–Gray model.

Variable	Strata	bDFS			PCSM			OS			MFS		
		HRa	95% CI	*p*	HRa	95% CI	*p*	HRa	95% CI	*p*	HRa	95% CI	*p*
Age	−70 vs. 71-	1.106	0.836–1.464	0.4817	0.6257	0.281–1.39	0.25	1.493	0.9374–2.377	0.091	0.7179	0.4392–1.173	0.1862
T Classification	≤2 vs. 3a≤	2.234	1.625–3.071	**<0.0001**	4.013	1385–11.62	**0.01**	1.95	1.169–3.254	**0.01057**	4.645	2.299–9.382	**<0.0001**
	≤3a vs. 3b≤	2.819	2.101–3.783	**<0.0001**	3.1887	1.454–6.988	**0.0038**	2.074	1.261–3.411	**0.004**	3.144	1.911–5.173	**<0.0001**
	≤3b vs. 4≤	4.837	2.855–8.195	**<0.0001**	5.699	1.632–19.9	**0.0064**	1.747	0.5496–5.552	0.3444	7.153	3.41–15	**<0.0001**
Gleason Score	≤7 vs. 8≤	1.652	1.227–2.224	**0.0009**	2.227	0.934–5.308	**0.0708**	1.794	1.097–2.933	**0.01989**	1.848	1.141–3.319	**0.0145**
	≤8 vs. 9≤	2.219	1.679–2.934	**<0.0001**	3.833	1.591–8.295	**0.0022**	1.978	1.257–3.114	**0.0032**	2.376	1.464–3.855	**0.0005**
	≤9 vs. 10≤	1.316	0.619–2.799	0.475	4.94	1.435–17.0	**0.011**	2.769	1.115–6.874	**0.0281**	1.82	0.571–5.801	0.3111
Pretreatment PSA (ng/mL)	≤20 vs. 20<	1.635	1.223–2.187	**0.0009**	1.393	0.6331–3.065	0.41	0.7676	0.4818–1.223	0.2655	1.508	0.9127–2.492	0.1088
	≤50 vs. 50<	2.001	1.482–2.701	**<0.0001**	1.774	0.7739–4.068	0.18	1.758	1.075–2.875	**0.02454**	2.074	1.242–3.461	**0.005**
	≤100 vs. 100<	1.621	1.097–2.576	**0.0171**	2.316	0.3038–17.65	0.42	1.135	0.520–2.475	0.7498	1.371	0.6254–3.006	0.4304
ADT duration (months)	≤40 vs. 41-	0.5067	0.372–0.689	**<0.0001**	0.9922	0.9746–1.01	0.39	1.193	0.7545–1.888	0.4497	0.8925	0.5434–1.466	0.6531
Modality	EBRT vs. BT ± EBRT	0.355	0.266–0.474	**<0.0001**	1.128	0.5181–2.467	0.76	0.9053	0.566–1.447	0.6775	0.9109	0.5537–1.498	0.7133
No. of very-high risk factors	VHR-0	1	(referent)	-	1	(referent)	-	1	(referent)	-	1	(referent)	-
	VHR-1	1.93	1.412–2.639	**<0.0001**	2.408	0.9297–6.244	**0.07**	1.8	1.092–2.966	**0.021**	1.41	0.795–2.502	0.24
	VHR-2	5.989	4.072–8.808	**<0.0001**	10.07	3.659–27.74	**<0.0001**	3.756	1.952–7.228	**<0.0001**	7.045	3.878–12.798	**<0.0001**

Competing risks regression model of prostate cancer-specific survival using the Fine–Gray model, treating other-cause mortality as a competing risk. BT = brachytherapy, EBRT = external beam radiotherapy VHRF-0, -1, and -2 indicate no, one, and two VHR factors, respectively. bDFS = biohemical failure-free survival, PCSM = prostate cancer specified mortality, OS = overall survival, MFSR = metastasis-free survival rate. Bold values indicate statistically significance.

**Table 4:** Multi-variate analysis for biochemical, overall, prostate cancer-specific, and metastasis-free survival rate using the Cox proportional hazards model or the Fine–Gray model.

Variable	Strata	bDFS			PCSM			OS			MFS		
		HRa	95% CI	*p*	HRa	95% CI	*p*	HRa	95% CI	*p*	HRa	95% CI	*p*
Age	≤70 vs. 71-	0.798	0.601–1.059	0.1182	0.6676	0.2844–1.567	0.35	1.572	0.982–2.518	0.0597	0.772	0.469–1.270	0.308
ADT Duration (months)	≤40 vs. 41-	0.87	0.606–1.250	0.4516	0.4535	0.1528–1.346	0.15	1.212	0.709–2.073	0.4823	0.825	0.466–1.463	0.5113
Modality	EBRT vs, BT ± EBRT	0.447	0.315–0.633	**<0.0001**	0.6297	0.2433–1.630	0.34	1.314	0.750–2.301	0.3401	1.53	0.876–2.888	0.1277
No. of very high-risk factors	VHR-0	1	(referent)	-	1	(referent)	-	1	(referent)	-	1	(referent)	-
	VHR-1	1.723	1.256–2.362	**0.0007**	2.391	0.8974–6.373	**0.081**	1.88	1.137–3.109	**0.0139**	1.491	0.837–2.656	0.1756
	VHR-2	4.55	3.065–6.755	**<0.0001**	9.607	3.29–28.05	**<0.0001**	4.327	2.206–8.487	**<0.0001**	7.904	4.251–14.696	**<0.0001**

Competing risks regression model was applied for prostate cancer-specific mortality using the Fine–Gray model, treating other-cause mortality as a competing risk. BT = brachytherapy, EBRT = external beam radiotherapy. VHRF-0, −1, and −2 indicate no VHRF, one VHRFs, and two VHRFs, respectively. bDFS = biohemical failure-free survival, PCSM = prostate cancer specified mortality, OS = overall survival, MFSR = metastasis-free survival rate. Bold values indicate statistically significance.

## Data Availability

The data of HDR-BT for this manuscript can be obtained from the public data base [7] and LDR-BT can be obtained from the author upon reasonable request.

## Supplementary Materials

The following are available online at https://www.mdpi.com/article/10.3390/cancers13143486/s1, Table S1: Detailed Treatment schedule.
